# Supplementary material for: PTM-Mamba: a PTM-aware protein language model with bidirectional gated Mamba blocks
Source: Nat Methods. 2025 Apr 10;22(5):945–9. doi: 10.1038/s41592-025-02656-9 (PMC12074982; doi:10.1038/s41592-025-02656-9)
Supplement: Supplementary file 1 — Supplementary Figs. 1–5, Codes 1–4 and Tables 1 and 2 [file 41592_2025_2656_MOESM1_ESM.pdf]

nature methods

Supplementary information

https://doi.org/10.1038/s41592-025-02656-9

PTM-Mamba: a PTM-aware protein language model with bidirectional gated Mamba blocks

In the format provided by the authors and unedited

Table of Contents

Supplementary Figures

1. PTM distributions in PTM-Mamba training set.
2. Length distribution of modified sequences in PTM-Mamba training dataset.
3. PTM-Mamba vs. PTM-Transformer training accuracy curve.
4. PTM-Mamba vs. baseline models on phosphorylation site prediction.
5. PTM-Mamba vs. baseline models on non-histone acetylation site prediction.

Supplementary Code

1. PyTorch representative code for classification head used for phosphorylation site prediction.
2. PyTorch representative code for classification head used for non-histone acetylation site prediction.
3. PyTorch representative code for classification head used for disease-association and druggability prediction.
4. PyTorch representative code for classification head used for prediction of PTM effect on protein-protein interactions.

Supplementary Tables

1. PTM tokens used in PTM-Mamba.
2. Hyperparameters used for embedding benchmarking tasks.

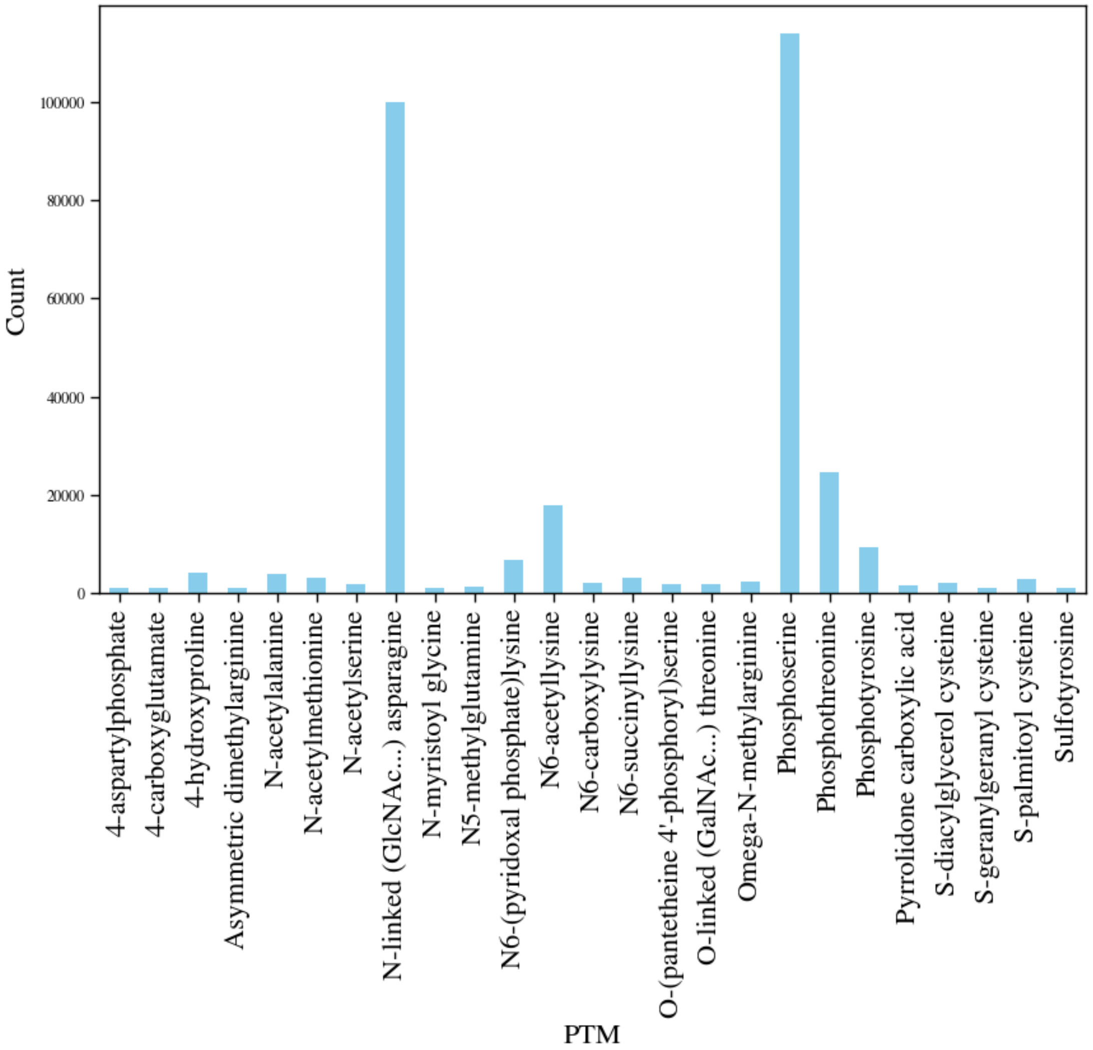

Supplementary Figure 1: PTM distributions in PTM-Mamba training set. A) The distribution of PTM annotations, demonstrating long-tail distributions. B) The distribution of the PTM sequence length, which is heavily centered at 400. A majority of PTM sequences are of length < 1500.

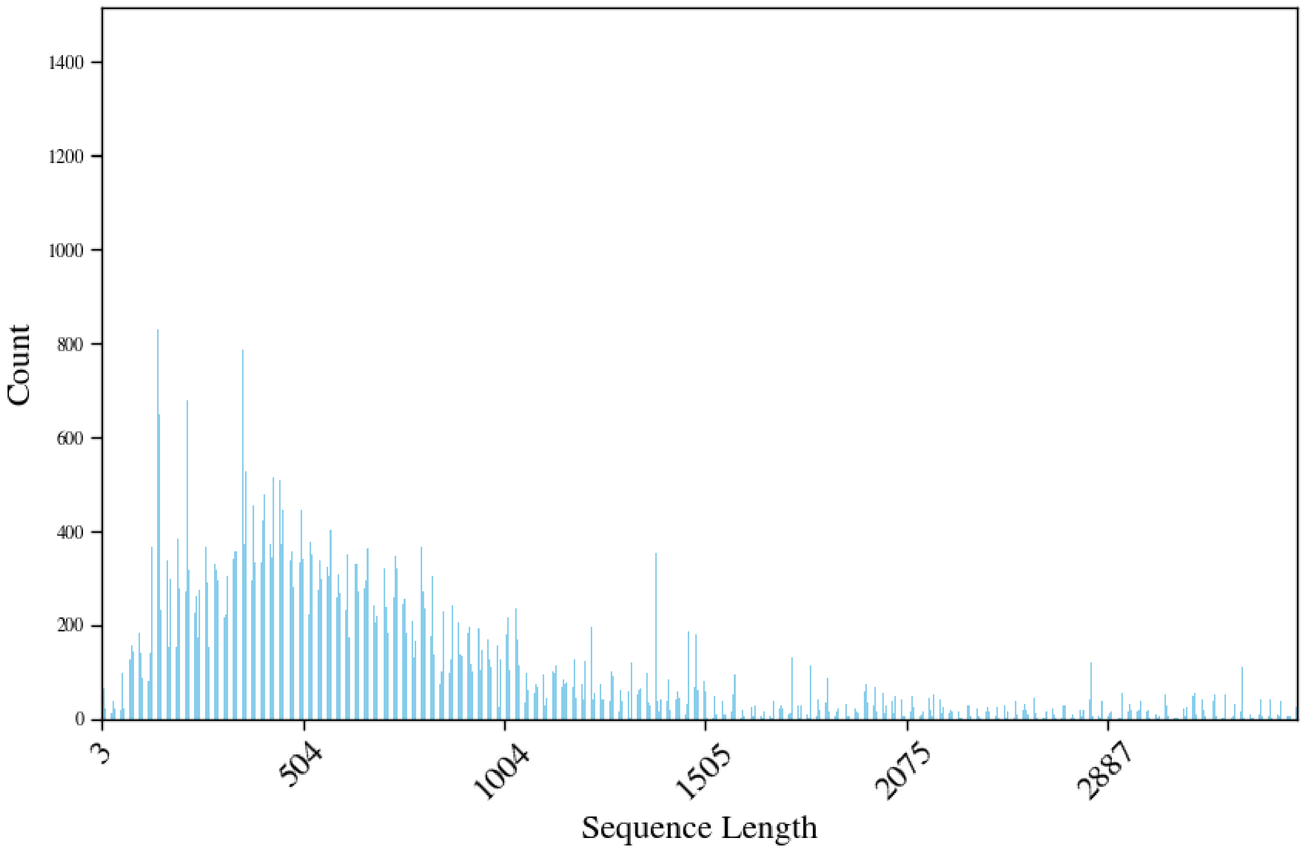

Supplementary Figure 2: Length distribution of modified sequences in PTM-Mamba training dataset.

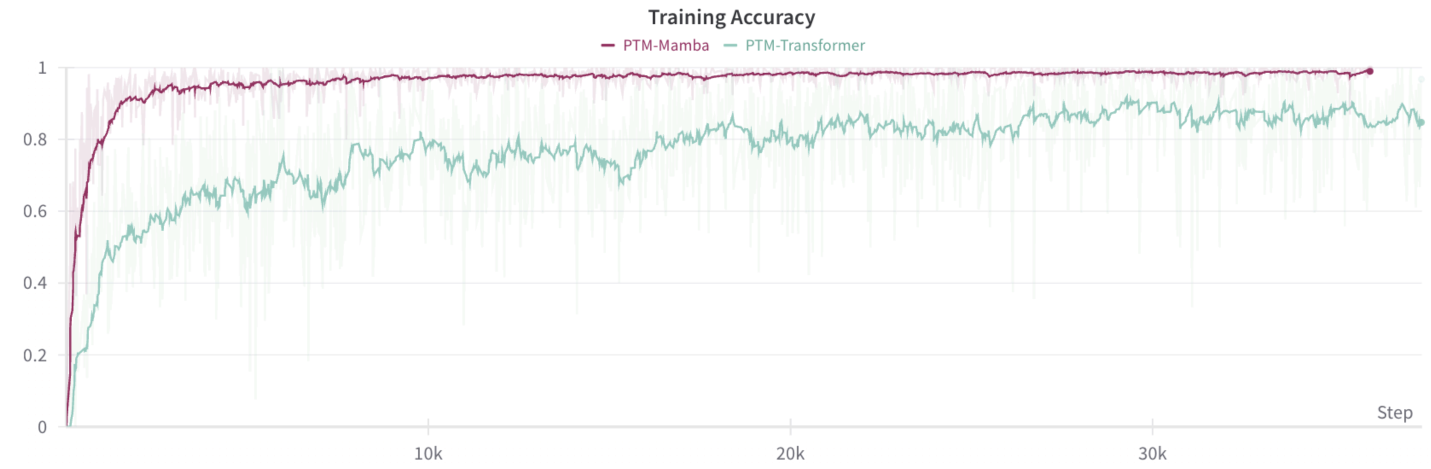

Supplementary Figure 3: PTM-Mamba vs. PTM-Transformer training accuracy curve. Iteration steps are shown on the x axis, and training accuracy on masked token prediction (0 to 1) are shown on the y axis.

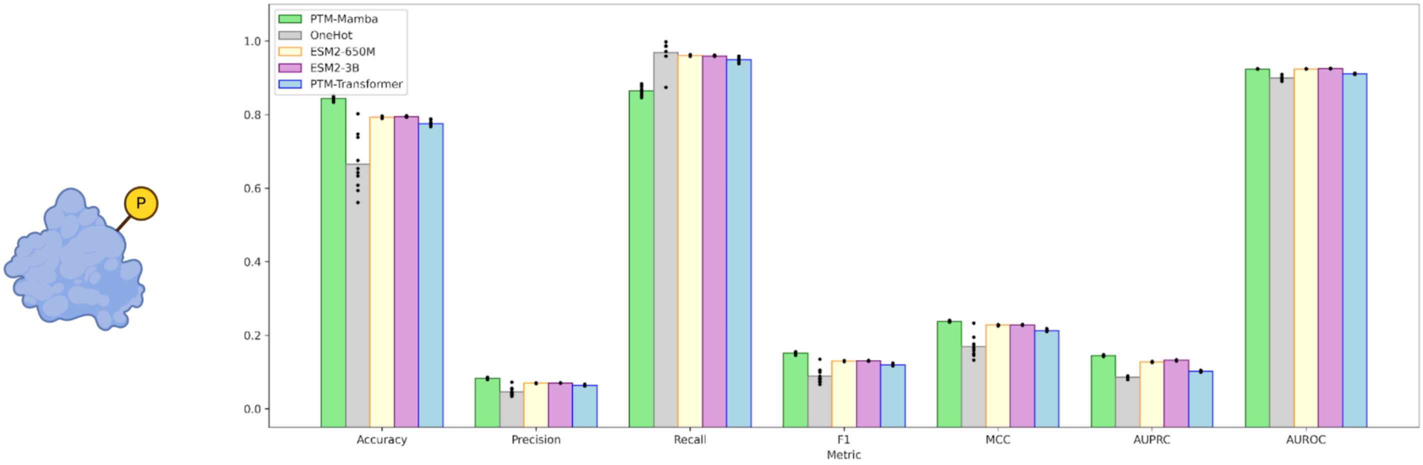

Supplementary Figure 4: PTM-Mamba vs. baseline models on phosphorylation site prediction. Training details can be found in the Methods section and representative code for the classification head can be found in Listing 1.

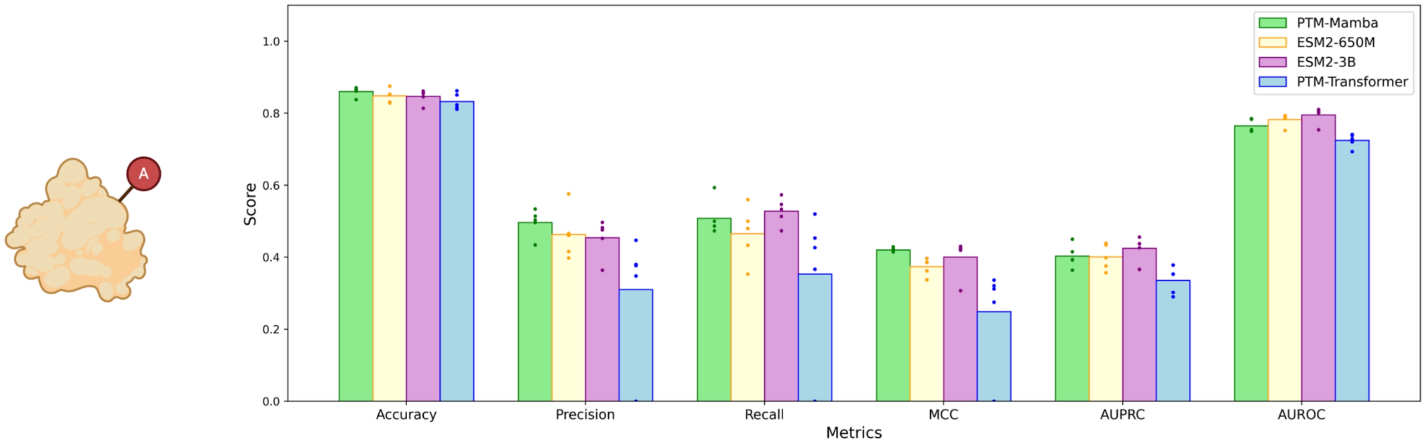

Supplementary Figure 5: PTM-Mamba vs. baseline models on non-histone acetylation site prediction. Training details can be found in the Methods section and representative code for the classification head can be found in Listing 2.

Supplementary Code 1: PyTorch representative code for classification head used for phosphorylation site prediction.

```python
class ClassificationHead(nn.Module):

    def __init__(self, hidden_size=128, num_labels=2, dropout=0.1):
        super().__init__()
        self.dense = nn.Linear(hidden_size, hidden_size)
        self.dropout = nn.Dropout(dropout)
        self.out_proj = nn.Linear(hidden_size, num_labels)

    def forward(self, features, **kwargs):
        x = features
        x = self.dropout(x)
        x = self.dense(x)
        x = torch.tanh(x)
        x = self.dropout(x)
        x = self.out_proj(x)
        return x
```

Supplementary Code 2. PyTorch representative code for classification head used for non-histone acetylation site prediction. The graph neural network transformer (GNNTrans) architecture is described in Meng, et al.[24]

```
class GNNTrans(nn.Module):
    def __init__(self, input_dim, hidden_dim, num_layers):
        super().__init__()
        self.num_layers = num_layers
        self.loss_func = nn.BCELoss()
        self.convs = torch.nn.ModuleList(
             [TransformerConv(in_channels=input_dim, out_channels=hidden_dim, heads=1)]
+
            [TransformerConv(in_channels=hidden_dim, out_channels=hidden_dim, heads=1)
                for _ in range(num_layers-1)]
        )

        self.mlp = nn.Sequential(
            nn.Dropout(p=0.5),
            nn.Linear(hidden_dim, hidden_dim),
            nn.ReLU(inplace=True),
            nn.Dropout(p=0.5),
            nn.Linear(hidden_dim, 64),
            nn.ReLU(inplace=True),
            nn.Dropout(p=0.3),
            nn.Linear(64, 1),
            nn.Sigmoid()
        )

    def get_conv_result(self, x, edge_index):
        for i in range(self.num_layers):
            x = self.convs[i](x=x, edge_index=edge_index)
            x = F.relu(x, inplace=True)
        return x

    def forward(self, data):
        x, edge_index, batch = data.emb, data.edge_index, data.batch
        idx = (data.ptr + int(len(data.seq[0]) / 2))[:-1]
        x = self.get_conv_result(x, edge_index)
        x = x[idx]
        out = self.mlp(x)
        return out
```

Supplementary Code 3: PyTorch representative code for classification head used for disease-association and druggability prediction.

```
class EmbeddingMLP(pl.LightningModule):
    def __init__(self, vocab_size, embedding_dim, lr=1e-3):
        super(EmbeddingMLP, self).__init__()
                            self.embedding    =    nn.Embedding(num_embeddings=vocab_size,
embedding_dim=embedding_dim)
        self.fc = nn.Sequential(
            nn.Linear(embedding_dim, 2)
        )
        self.lr = lr

    def forward(self, x):
        x = x.to(torch.int32)
        embedded = self.embedding(x)
        embedded = embedded.mean(dim=1)
        linear_output = self.fc(embedded)
        softmax_output = F.log_softmax(linear_output, dim=1)
        return softmax_output
```

Supplementary Code 4: PyTorch representative code for classification head used for prediction of PTM effect on protein-protein interactions.

```
class TransformerClassifier(nn.Module):
    def __init__(self, dropout_rate=0.3, max_length=2000):
        super(TransformerClassifier, self).__init__()

        self.fc = nn.Sequential(
            nn.Linear(2 * max_length, max_length),
            nn.ReLU(),
            nn.Dropout(dropout_rate),
            nn.Linear(max_length, 1)
        )

    def forward(self, binder, wt, ptm):
        binder_wt = torch.cat([binder, wt], dim=-1)
        binder_ptm = torch.cat([binder, ptm], dim=-1)

        x = self.fc(binder_wt - binder_ptm)
        return x.squeeze(-1)
```

Supplementary Table 1. PTMs specifically represented in PTM-Mamba as new tokens.

PTM Token	Annotation
N-linked (GlcNAc...) asparagine	Addition of N-acetylglucosamine to asparagine residues
Pyrrolidone carboxylic acid	Formation of a lactam ring from the N-terminal glutamine
Phosphoserine	Addition of a phosphate group to serine residues
Phosphothreonine	Addition of a phosphate group to threonine residues
N-acetylalanine	Acetylation of the N-terminal alanine
N-acetylmethionine	Acetylation of the N-terminal methionine
N6-acetyllysine	Acetylation of the lysine residue at the ε-amino group
Phosphotyrosine	Addition of a phosphate group to tyrosine residues
S-diacylglycerol cysteine	Attachment of diacylglycerol to cysteine residues
N6-(pyridoxal phosphate)lysine	Addition of pyridoxal phosphate to lysine residues
N-acetylserine	Acetylation of the serine residue
N6-carboxylysine	Carboxylation of the lysine residue
N6-succinyllysine	Succinylation of the lysine residue
S-palmitoyl cysteine	Palmitoylation of cysteine residues
O-(pantetheine 4-phosphoryl)serine	Addition of pantetheine phosphate to serine residues
Sulfotyrosine	Sulfation of tyrosine residues
O-linked (GalNAc...) threonine	Addition of N-acetylgalactosamine to threonine residues
Omega-N-methylarginine	Methylation of the arginine residue
N-myristoyl glycine	Myristoylation of glycine residues
4-hydroxyproline	Hydroxylation of proline residues
Asymmetric dimethylarginine	Dimethylation of the arginine residue in an asymmetric manner
N5-methylglutamine	Methylation of the glutamine residue
4-aspartylphosphate	Addition of a phosphate group to aspartate residues
S-geranylgeranyl cysteine	Attachment of geranylgeranyl to cysteine residues
4-carboxyglutamate	Carboxylation of glutamate residues

Supplementary Table 2. Hyperparameters used for embedding benchmarking tasks.

Benchmarking Task	Optimizer	Learning Rate	# of Epochs	Batch Size
Phosphorylation Site Prediction	Adam	0.001	3	256
NHAC Site Prediction	Adam	0.00003	100	64
Druggability Prediction	Adam	0.001	2	1
Disease-Association Prediction	Adam	0.001	2	1
PTM Effect on PPI Prediction	AdamW	0.001	30	16
