# Supplementary material for: PTM-Mamba: a PTM-aware protein language model with bidirectional gated Mamba blocks
Source: Nat Methods. 2025 Apr 10;22(5):945–9. doi: 10.1038/s41592-025-02656-9 (PMC12074982; doi:10.1038/s41592-025-02656-9)
Supplement: Supplementary file 2 — Reporting Summary [file 41592_2025_2656_MOESM2_ESM.pdf]

nature portfolio

Corresponding author(s): Pranam Chatterjee

Last updated by author(s): Feb 3, 2025

Reporting Summary

Nature Portfolio wishes to improve the reproducibility of the work that we publish. This form provides structure for consistency and transparency in reporting. For further information on Nature Portfolio policies, see our Editorial Policies and the Editorial Policy Checklist.

Statistics

For all statistical analyses, confirm that the following items are present in the figure legend, table legend, main text, or Methods section.

n/a	Confirmed	
☐	☒	The exact sample size (*n*) for each experimental group/condition, given as a discrete number and unit of measurement
☐	☒	A statement on whether measurements were taken from distinct samples or whether the same sample was measured repeatedly
☒	☐	The statistical test(s) used AND whether they are one- or two-sided<br>*Only common tests should be described solely by name; describe more complex techniques in the Methods section.*
☒	☐	A description of all covariates tested
☒	☐	A description of any assumptions or corrections, such as tests of normality and adjustment for multiple comparisons
☐	☒	A full description of the statistical parameters including central tendency (e.g. means) or other basic estimates (e.g. regression coefficient) AND variation (e.g. standard deviation) or associated estimates of uncertainty (e.g. confidence intervals)
☒	☐	For null hypothesis testing, the test statistic (e.g. *F*, *t*, *r*) with confidence intervals, effect sizes, degrees of freedom and *P* value noted<br>*Give P values as exact values whenever suitable.*
☒	☐	For Bayesian analysis, information on the choice of priors and Markov chain Monte Carlo settings
☒	☐	For hierarchical and complex designs, identification of the appropriate level for tests and full reporting of outcomes
☒	☐	Estimates of effect sizes (e.g. Cohen's *d*, Pearson's *r*), indicating how they were calculated

Our web collection on statistics for biologists contains articles on many of the points above.

Software and code

Policy information about availability of computer code

Data collection

Model training data was curated from UniProt15. Specifically, 311,350 experimentally-validated PTM records were collected from Swiss-Prot and mapped the PTM annotations of their protein to their respective sequences to construct the new PTM sequences. The final dataset includes a total of 79,707 PTM sequences. Data curation code can be found at: https://github.com/programmablebio/ptm-mamba/tree/main/ptm_data_preprocessing.

Datasets for the four benchmarking tasks were collected from the following sources. Phosphorylation site data was obtained from the corresponding ProteinBERT benchmark,25 originally derived from PhospoSitePlus18 and filtered for sequences between 256 and 512 amino acids in length, yielding a training set of 15,588 sequences, a validation set of 1707 sequences, and a testing set of 3106 sequences. Non-histone acetylation site prediction was performed equivalently as described in prior literature, using the NHAC (Non-Histone Acetylation Collection) dataset.26 The druggability and disease association datasets were curated from the dbPTM database.17 PPI data describing the effect of PTMs were curated from PTMint, which encompasses 2,477 non-redundant PTM sites in 1169 proteins affecting 2371 protein-protein pairs. Briefly, wild-type sequences were mapped to corresponding entries in the PTM-Mamba dataset, and wild-type residues were replaced by the corresponding, position-specific PTMs for tokenization by specified models. For all other baseline models trained with standard one-hot embeddings or ESM-2 embeddings, the corresponding wild-type sequence was used as input.

Data analysis

For all the benchmark tasks, we leverage the embeddings from pretrained PTM-Mamba and ESM-2 models and finetune a classification head on top of the embeddings. We extensively tuned the classification head architectures as well as the training hyperparameters for the benchmarks and have reported the optimal settings in Supplementary Listing 1-4 and Supplementary Table 2. For models trained on one-hot embeddings of wild-type input sequences, an nn.Embedding layer followed by a linear layer was utilized. All benchmark models were trained on a Nvidia 8xA100 GPU DGX system with 640 GB of shared VRAM. For robust performance comparison, we replicate each model (n=5) and

report the individual and average results. Models were evaluated using the Accuracy, Precision, Recall, F1 Score, MCC, AUROC, and AUPRC metrics via scikit-learn.27

For manuscripts utilizing custom algorithms or software that are central to the research but not yet described in published literature, software must be made available to editors and reviewers. We strongly encourage code deposition in a community repository (e.g. GitHub). See the Nature Portfolio guidelines for submitting code & software for further information.

Data

Policy information about availability of data

All manuscripts must include a data availability statement. This statement should provide the following information, where applicable:

- Accession codes, unique identifiers, or web links for publicly available datasets
- A description of any restrictions on data availability
- For clinical datasets or third party data, please ensure that the statement adheres to our policy

All data needed to evaluate the conclusions are presented in the paper, tables, and supplementary information, and are further available at https://doi.org/10.5281/zenodo.14794992.

Human research participants

Policy information about studies involving human research participants and Sex and Gender in Research.

Reporting on sex and gender	N/A
Population characteristics	N/A
Recruitment	N/A
Ethics oversight	N/A

Note that full information on the approval of the study protocol must also be provided in the manuscript.

Field-specific reporting

Please select the one below that is the best fit for your research. If you are not sure, read the appropriate sections before making your selection.

☒ Life sciences ☐ Behavioural & social sciences ☐ Ecological, evolutionary & environmental sciences

For a reference copy of the document with all sections, see nature.com/documents/nr-reporting-summary-flat.pdf

Life sciences study design

All studies must disclose on these points even when the disclosure is negative.

Sample size	Sample size determination was not established. We chose sample sizes based on data availability and number of models required for benchmarking.
Data exclusions	No data was excluded.
Replication	All benchmarks in panels A-C were performed in replicates (n = 5). All attempts at replication were successful.
Randomization	Random seeds were used to prevent overfitting.
Blinding	No blinding was necessary for this study. As a computational study, the training models must be known to the researcher to accurately evaluate results.

Reporting for specific materials, systems and methods

We require information from authors about some types of materials, experimental systems and methods used in many studies. Here, indicate whether each material, system or method listed is relevant to your study. If you are not sure if a list item applies to your research, read the appropriate section before selecting a response.

Materials & experimental systems

n/a	Involved in the study
☒	☐ Antibodies
☒	☐ Eukaryotic cell lines
☒	☐ Palaeontology and archaeology
☒	☐ Animals and other organisms
☒	☐ Clinical data
☒	☐ Dual use research of concern

Methods

n/a	Involved in the study
☒	☐ ChIP-seq
☒	☐ Flow cytometry
☒	☐ MRI-based neuroimaging
